# Exploration of weekly variation in naloxone possession and carriage among people who use opioids in New York City before, during, and after the COVID-19 pandemic

**DOI:** 10.1371/journal.pone.0307151

**Published:** 2024-07-18

**Authors:** Alexis M. Roth, Kathleen M. Ward, Devon J. Hensel, Luther Elliott, Alex S. Bennett

**Affiliations:** 1 Department of Community Health and Prevention, Drexel University Dornsife School of Public Health, Philadelphia, PA, United States of America; 2 Department of Pediatrics, Indiana University School of Medicine, Indianapolis, IN, United States of America; 3 Department of Sociology, Indiana University Indianapolis, Indianapolis, IN, United States of America; 4 Center for Drug Use and HIV/HCV Research, New York University School of Global Public Health, New York, NY, United States of America; Lahore College for Women University, PAKISTAN

## Abstract

**Background:**

Naloxone is critical for reversing opioid-related overdoses. However, there is a dearth of research examining how naloxone possession and carriage are impacted by time-varying individual and social determinants, and if this differed during the height of the COVID-related mitigation measures (e.g., shutdowns).

**Methods:**

We utilized weekly ecological momentary assessments (EMA) to measure factors associated with naloxone possession and carriage among 40 people who use illicit opioids in New York City, for 24 months. Descriptive statistics were used to explore the frequency of weeks with consistent naloxone possession and carriage. Mixed effects binary and multivariable logistic regression was used to test for the impact of time-varying EMA- and baseline-level factors on each outcome.

**Results:**

Approximately 70% of weekly EMAs were associated with consistent naloxone possession or carriage. In multivariable models, compared to during the height of the COVID-related shutdowns (March 12, 2020-May 19, 2021), the time before was associated with lower odds of consistent possession (Odds Ratio (OR) = 0.05, 95% Confidence Interval (CI) = 0.01–0.15) and consistent carriage (OR = 0.06, CI = 0.01–0.25). Additionally, being female (OR = 11.15, CI = 2.85–43.42), being White versus being Black or Hispanic/Latinx (OR = 8.05, CI = 1.96–33.06), and lifetime overdose (OR = 1.96, CI = 1.16–19.80) were associated with higher odds of consistent possession. Recent opioid injection (OR = 3.66, CI = 1.34–9.94), being female (OR = 7.91, CI = 3.91–8.23), and being White (OR = 5.77, CI = 1.35–24.55) were associated with higher odds of consistent carriage. Not wanting to be perceived as a drug user was reported in nearly one third (29.0%; 190/656) of EMAs where inconsistent possession was reported.

**Conclusions:**

Our findings paint a relatively positive picture of possession and carriage during COVID-related shutdowns, particularly among white and female participants, and highlight the importance of capturing time-varying factors to understand naloxone-related behavior. To curb growing disparities, outreach to equip Black and Hispanic/Latinx people with naloxone is needed as well as interventions to reduce stigma as a barrier to naloxone engagement.

## Introduction

In the United States, preventable opioid-related overdose morbidity and mortality remains a critical public health challenge [1]. Since 2013, the soaring rate of opioid-related fatal overdoses has been attributed to the emergence of illicit synthetic opioids (e.g., fentanyl and fentanyl analogs) in markets formerly dominated by heroin [2]. For the first time, in 2021, there were more than 100,000 overdose deaths nationwide in a single year [1]. Opioid-related overdose mortality in New York City (NYC), including Manhattan and surrounding boroughs (Bronx, Queens, Brooklyn, and Staten Island), has mirrored national trends. In 2021, NYC reported more overdose-related emergency department visits, hospitalizations, and administrations of the opioid overdose reversal agent, naloxone, than ever before [3]. In 2022, the upward trend of fatal overdoses continued, increasing by 12%, in the wake of the COVID-19 pandemic [4–7].

Nationally, and in NYC, overdose prevention efforts have focused on policies and programs that increase community member access to naloxone. Since 1996, the number of overdose education and naloxone distribution programs has grown [8]. Research shows these programs reach individuals at high risk for overdose, equip them with naloxone, and are a cost-effective strategy for reducing opioid-related overdose deaths [9, 10]. Increasing the availability of naloxone, especially to people who use opioids (PWUO), results in rapid and dramatic increases in overdose reversals [9]. In a study of Massachusetts naloxone outcomes, 87% of rescue attempts involving naloxone were made by PWUO themselves [11]. Yet, community coverage of naloxone continues to lag well behind documented need [12], which may partially explain why a recent systematic review found only moderate levels of naloxone possession (57%, 95% Confidence Interval (CI) = 47–67%) and low naloxone carriage (20%, CI = 12–31%) among people who use drugs worldwide [13].

In the context of the COVID-19 pandemic, there was a surge in fatal overdoses in the US, with the largest increase occurring between March and May 2020 [14]. Recommended COVID-19 mitigation measures (e.g., shutdowns, stay at home orders, and physical distancing recommendations) forced closure and/or decreased capacity of harm reduction organizations (e.g., syringe services programs [SEPs] and drug treatment centers). Mitigation measures also resulted in disruptions to the local drug supplies and limited bystander presence for overdose reversal. Taken together, these factors were chief among the drivers of overdose during this time [15–17]. While scholars have highlighted the need to better understand the effects of COVID-19 and other contextual factors on overdose risk, this literature is still emerging.

More research is needed to understand the impact of COVID-19 on naloxone possession and carriage. However, heterogeneity in how these behaviors are measured limits our ability to compare outcomes across studies [13]. For example, *“have*,*” “own*,*” “possess”* and *“carry”* are often used, in reference to naloxone, though the meaning of each is slightly different. Further, reference periods vary, with some studies operationalizing these behaviors as a proportion of the time (e.g., always to never) over various recall windows (e.g., past 7 days to past 3 months), as a simple yes/no in the here and now, or, seldomly, as immediate access each time an opioid-related overdose is witnessed [13]. Overall, cross-sectional approaches, which measure naloxone-related behavior at a single timepoint (e.g., *ever* received or *currently* possess naloxone), do not provide the repeated data collection structure needed to accurately gauge a behavior that changes over relatively short amounts of time and by context [18].

Ecological momentary assessment (EMA), which emerged in empirical studies of substance use in the mid-1990s, has untapped potential for improving our understanding of naloxone-related behavior. Studies employing EMA frequently assess an individual’s behaviors and experiences as they go about their life in their own environment. This approach has been shown to decrease recall bias and improve ecological validity [19]. While EMA has been utilized to study various sensations and behavior that are influenced by context (e.g., drug craving, drug relapse, and condom non-use), it has yet to be applied to naloxone [20, 21].

In this study, we engaged weekly EMA to measure how often naloxone is possessed and carried across time and to explore the individual and/or structural factors associated with consistent possession/carriage. Participants were PWUO (n = 40) living in NYC, all of whom were trained as overdose responders and equipped with intranasal naloxone. We used the response horizon of the COVID-19 pandemic to recategorize time into three eras–the before times, the height of the mitigation measures (shutdowns), and the period after shutdowns, when the city began to reopen. This allowed us to explore the influence of COVID-19 on naloxone possession and carriage in NYC over time.

## Methods

Participants (n = 40) in the EMA study represent a purposively selected subsample from a larger cohort study of 575 individuals who were aged 18 or older, currently using non-prescribed opioids (including heroin, fentanyl, and prescription opioids used without prescription, verified using rapid urinalysis) and resided in one of the 5 boroughs of NYC [22]. Participants were enrolled and followed for a period of 24 months. To identify participants eligible for the EMA study, research staff kept a log containing key demographic information (ethnicity, age, gender), substance use by drug and route of administration, overdose experiences, naloxone exposure (used on others or self), and whether participants were comfortable using text messaging and email. Participants were sequentially recruited until 40 individuals were enrolled, with roughly 50% being people who inject drugs, at least 50% reporting a past overdose experience, and proportionally represented substance use patterns, naloxone exposures, race, gender, and ethnicity. All participants were trained as overdose responders and equipped with intranasal naloxone during the baseline enrollment process.

### Data collection

From May 1, 2019 to May 30, 2022, enrolled participants received four EMAs per month at semi-randomized times (e.g., randomized hours between 11am and 9pm with targeted ratios of weekend to weekday days). Each week, they received an automated SMS message that directed them to a data collection portal to complete the EMA comprised of a brief set of questions about their most recent 24-hour period of opioid use. Participants were compensated $15 for every weekly EMA entry they completed, in addition to honorariums provided by the larger cohort study. All human research protocols were approved by the New York University Grossman School of Medicine’s Institutional Review Board and all participants provided written informed consent.

### Measures

#### Outcome variables

Naloxone possession was measured with a single EMA item, “In the past 7 days, how often have you had a naloxone kit in your personal possession?” and naloxone carriage was a single EMA item, “In the past 7 days, when you’ve been outside of your current residence, how often have you carried naloxone?” The response options, all the time, most of the time, rarely and never, were dichotomized as *inconsistent* [most of the time, rarely and never] vs. *consistent* [all the time] for analysis.

#### EMA-level factors

EMA variables included: the date the EMA entry was made, recategorized as three time periods: *before COVID-related shutdowns* (before March 11, 2020), *during the height of the COVID-related shutdowns* (March 12, 2020 to May 19, 2021) or *after the COVID-19 shutdowns* (May 20, 2021 to May 31, 2022) [23–25]. *Positive affect* (feeling happy and joyful; scale α = 0.91) and *negative affect* (feeling nervous, anxious, stressed, overwhelmed, depressed, sad, angry, frustrated, angry with yourself, and guilty; scale α = 0.94) were measured on a 4-point scale from “not at all” to “extremely”. Other variables included a *stress and control scale* [26], *pain scale* (single visual analogue scale: 0–10), *current drug craving* (no/yes for 23 substances; summed all “yes” answers), *any past 24 hour opioid use* (categorized as no opioids used, Buprenorphine or Methadone only, and any illegal opioids with or without other drug use; taken from past 24 hour use inventory [all no/yes] of 23 substances), and *any past week opioid injection* (no vs. yes [ref]).

#### Baseline factors

Baseline factors included: *current age* (centered at 18 years for analysis), *gender* (male vs. female [ref]), *race* (White [ref] vs. Black or Hispanic/Latinx); *currently homeless* (no vs. yes [ref]), *frequency of Syringe Exchange Program use* (“How often have you visited a SEP in the last 3 months?” measured with a 5-point scale ranging from “Never” to “Daily or Almost Daily”), *any lifetime overdose* (no vs. yes [ref]) and *number of people in network who used opioids*.

#### Descriptive analysis variables

Because we were interested in why naloxone was not possessed or carried; we conducted a supplementary analysis to further contextualize these instances. Participants who endorsed inconsistent possession in the last 7 days were asked *“What explains why you have not had naloxone in your possession*?*”* and prompted to select one: *It was lost or stolen and I haven’t refilled it yet; I used it and I haven’t refilled it yet; I don’t want to be perceived as a drug user; It was confiscated and I haven’t refilled it yet; I gave it or lent it to someone; Other*. Participants who endorsed inconsistent carriage in the last 7 days were asked “What were your reasons for not always carrying naloxone with you when you were outside of your residence?” and prompted to select all that apply: *I forgot to carry it with me; I wasn’t around people who use opioids; I didn’t want to be responsible for other people; I didn’t want to take it where I was going; I’m tired of thinking about overdose; Other*.

### Statistical approach

We first used descriptive statistics to assess the frequency of weekly EMA entries that were associated with consistent and inconsistent naloxone possession and carriage. Next, we used mixed effects logistic regression (Stata, v.17) to evaluate the impact of each EMA- and baseline-level factor on each outcome variable. A random intercept approach allowed our estimates to vary across individuals and adjusted estimates for multiple EMAs contributed by each participant. We conducted bivariate models, in which the association of each factor with variables that were significant at p<0.10 were retained for estimation in a final multivariable model. As part of a sensitivity analysis, we investigated a “time-varying lagged” effect of opioid injection (*any opioid injection the week before last* (no vs. yes [ref])) on naloxone possession and carriage. Finally, we used descriptive statistics to assess the frequency of each reason why naloxone was *not* possessed or carried.

## Results

### Analytic sample

Forty participants were enrolled in the EMA study. For this analysis, we eliminated three individuals who reported no opioid use in the past seven days. The 37 participants in the analytic sample submitted 2320 EMAs. The average number of weekly EMAs submitted per participant in total was 76.1 (SD = 35.6). An average of 18.4 EMAs (SD = 14.7) were submitted in the period before the COVID-related shutdowns, 49.9 (SD = 12.9) were submitted during and 22.6 (SD = 17.1) entries were submitted after. Participant characteristics are shown in **Table 1**.

**Table 1:** Participant demographics (N = 40).

Characteristic	% (N) or (Mean (SD); Median, Min/Max)
**Age**	35.92 (10.52); 35, 20/62
**Gender** (n = 37)	
Male	47.5 (19)
Female	45.0 (18)
Other	7.5 (3)
**Sexual orientation** (n = 36)	
Heterosexual	74.3 (29)
Gay/Lesbian	7.6 (3)
Bisexual	15.3 (6)
Other	2.5 (1)
**Race** (n = 34)	
American Indiana/Alaska Native	5.0 (2)
White	64.5 (25)
Black/African American	20.0 (8)
**Hispanic or Latinx (yes)** (n = 37)	45.0 (18)
**Marital status** (n = 37)	
Never married	62.5 (25)
Married/Living together as married	22.5 (9)
Separated/Divorced/Widowed	15.0 (6)
**Highest education level** (n = 37)	
Less than HS	22.5 (9)
HS degree or GED	32.5 (13)
Any college	45.0 (18)
**Current employment status** (n = 37)	
Unemployed	47.5 (19)
Unable to work for health reasons or retired	22.5 (9)
Formal employment (full or part time)	20.0 (8)
Off-books, informal cash jobs	10.0 (4)
**Any past year sex work (yes)** (n = 37)	17.5 (7)
**Ever been homelessness (yes)** (n = 37)	77.5 (31)
**Currently homeless (yes)** (n = 37)	40.0 (16)
**Age of first opioid use** (n = 37)	20.1 (5.8); 18, 12/34
**Ever overdose (yes)** (n = 37)	60.0 (24)
**Ever inject opioids (yes) **(n = 37)	80.0 (29)
**Ever regularly binge drink alcohol (yes)** (n = 37)	35.0 (14)
**Trained to use naloxone prior to study participation** (n = 37)	67.5 (25)
**Injected during EMA study** (n = 37)	54.0 (20)
**Ever in prison (yes)** (n = 37)	38.8 (14)
**Household income** (n = 36)	
$0-$9,999	58.9 (23)
$10,000+	40.0 (16)
**Have health insurance (yes)** (n = 37)	97.5 (36)
**Visited syringe exchange program in past 3 months** (n = 37)	
Never	45.0 (18)
Monthly	25.0 (10)
Weekly	15.0 (6)
More than weekly	15.0 (6)

### Naloxone possession and carriage

As shown in **Table 2**, approximately 70% of weekly EMAs were associated with consistent possession or carriage.

**Table 2:** EMA reported frequency of naloxone possession and carriage.

Outcome	Statistic
**Individual-level (N = 40)**	*Mean (SD); Median (Minimum/Maximum)*
Average number of weeks with consistent naloxone possession	47.5 (38.1); 52 (0/116)
Average number of weeks with consistent naloxone carriage	45.0 (38.4); 52 (0/118)
**EMA-level (N = 2320)**	*Percent of EMAs (N)*
Naloxone possession	
vConsistently (all of the time)	70.5% (1637)
Never	6.2% (144)
Rarely	7.8% (182)
Most of the time	15.4% (357)
Naloxone carriage	
Consistently (all of the time)	67.7% (1571)
Never	5.6% (131)
Rarely	6.9% (161)
Most of the time	19.7% (457)

#### Naloxone possession

In bivariate models, compared to the time during the COVID-related shutdowns (March 12, 2020 to May 19, 2021), the time before the COVID-related shutdowns (May 1, 2019 to March 11, 2020) was associated with lower odds of consistent naloxone possession (OR = 0.11, CI = 0.06–0.18). In addition, higher mean craving (OR = 0.97, CI = 0.94–0.99) was associated with lower odds of consistent naloxone possession. Any opioid injection in the past seven days was associated with higher odds of consistent naloxone possession (OR = 2.28, CI:1.26–4.10).

Meanwhile, compared to during the COVID-related shutdowns, the time after COVID-related shutdowns (May 20, 2021 to May 31, 2022) was associated with higher odds of consistent possession (OR = 2.04, CI = 1.14–3.65). Additionally, being female (OR = 6.95, CI = 2.21–21.82), being White (OR = 6.41, CI = 2.07–19.82), having a higher frequency of SEP use (OR = 2.40, CI = 1.26–4.58), and any lifetime overdose (OR = 7.42, CI = 2.54–21.67) were linked to greater odds of consistent naloxone possession (**Table 3**).

**Table 3:** Weekly EMA- and baseline-level factors associated with consistent naloxone possession and carriage.

	Consistent Naloxone Possession		Consistent Naloxone Carriage	
Weekly EMA Factors	Bivariate	Multivariable	Bivariate	Multivariable
COVID-19 (ref: during)				
Before	**0.11 (0.06–0.18)** ***	**0.05 (0.01–0.15)** ***	**0.17 (0.11–0.30)** ***	**0.06 (0.01–0.25)** ***
After	**2.04 (1.14–3.65)** ***	2.72 (0.33–2.25)	1.15 (0.68–1.92)	0.79 (0.14–4.35)
Positive affect	1.03 (0.93–1.13)		0.99 (0.88–1.19)	
Negative affect	1.01 (0.98–1.04)		0.99 (0.96–1.03)	
Stress	0.96 (0.91–1.02)		1.01 (0.94–1.08)	
Pain	1.05 (0.94–1.18)		1.01 (0.89–1.12)	
Craving	**0.97 (0.94–0.99)** *	0.89 (0.65–1.14)	**0.95 (0.92–0.98)** **	0.95 (0.72–1.25)
Past 24-hour opioid use				
No Opioids, regardless of other use (ref)	--		--	
Bupe or Methadone only	0.82 (0.38–1.75)		0.52 (0.23–1.14)	
Illegal opioids w/ or w/o other drug use	1.72 (0.99–2.99)		1.45 (0.83–2.35)	
Injected in past 7 days (yes)	**2.28 (1.26–4.10)** **	2.27 (0.92–5.46)	**2.00 (1.12–3.57)** *	**3.66 (1.34–9.94)** **
**Baseline Factors**				
Current age (yrs: centered at 18)	0.91 (0.83–1.01)		0.90 (0.80–1.01)	
Gender (female)	**6.95 (2.21–21.82)** **	**11.15 (2.85–43.42)** **	**15.01 (3.95–56.95)** ***	**7.91 (3.91–8.23)** ***
White (yes)	**6.41 (2.07–19.82)** **	**8.05 (1.96–33.06)** **	**5.39 (1.50–19.39)** *	**5.77 (1.35–24.55)** **
Currently homeless (yes)	2.25 (0.58–8.70)		2.48 (0.56–10.95)	
Frequency of SEP use	**2.40 (1.26–4.58)** **	0.67 (0.29–1.66)	**2.55 (1.20–5.41)** *	0.54 (0.21–1.38)
Ever overdose in lifetime (yes)	**7.42 (2.54–21.67)** ***	**4.80 (1.16–19.80)** *	**9.13 (2.42–34.33)** **	1.87 (0.44–7.91)
Network size (continuous)	0.98 (0.95–1.01)		0.98 (0.95–1.01)	

OR = Odds Ratio; 95% CI = 95% Confidence Interval

*p < .05

**p < .01

***p < .001

In multivariable models, being female (OR = 11.15, CI = 2.85–43.42), being White (OR = 8.05, CI = 1.96–33.06) and lifetime overdose (OR = 4.80, CI = 1.16–19.80) were associated with higher odds of consistent possession. In addition, the time period before COVID-related shutdowns was associated with lower odds of consistent possession compared to during COVID-related shutdowns (OR = 0.05, CI = 0.01–0.15).

Participants reported not possessing naloxone in less than 30% of EMAs (683/2320) and provided a rationale for nearly most of these weeks (96%, 656/683). Not wanting to be perceived as a drug user (29.0%; 190/656), giving or lending it to someone else (19.4%; 127/656), failing to refill after use (15.2%; 100/656), or failing to refill after it was lost or stolen (13.7%; 90/656) were the most common reasons for not possessing naloxone.

#### Naloxone carriage

In bivariate models, compared to during the COVID-related shutdowns (March 12, 2020 to May 19, 2021), the time before COVID-related shutdowns (May 1, 2019 to March 11, 2020) was associated with lower odds of consistent carriage (OR = 0.17, CI = 0.11–0.30). Higher mean craving (OR = 0.95, CI = 0.92–0.98) was associated with lower odds of consistent carriage. In contrast, any opioid injection in the past seven days (OR = 2.00, CI = 1.12–3.57), being female (OR = 15.01, CI = 3.95–56.95), being White (OR = 5.39, CI = 1.50–19.39), higher frequency of SEP use (OR = 2.55, CI = 1.20–5.41) and any lifetime overdose (OR = 9.13, CI = 2.42–34.33) were linked to higher odds of consistent carriage (**Table 3**).

In multivariable models, opioid injection in the past seven days (OR = 3.66, CI = 1.34–9.94), being female (OR = 7.91, CI = 3.91–8.23), and being White (OR = 5.77, CI = 1.35–24.55) were associated with higher odds of consistent carriage. In contrast, the time before COVID-related shutdowns (OR = 0.06, CI = 0.01–0.25) was associated with lower odds of consistent carriage.

Participants reported not carrying naloxone in less than 33% of EMAs (749/2320) and provided a rationale for about half of these weeks (381/749). Non-carriage was most often attributed to forgetting to carry it (39.9%;152/381), not being around people who use opioids (31.8%, 121/381), and not wanting to take it where they were going (21.5%, 82/381). Participants rarely endorsed not wanting to be responsible for other people (2.6%, 14/381) or being tired of thinking about overdose (2.8%, 11/381) as their rationale for non-carriage.

#### Sensitivity analysis

In a sensitivity analysis, there was no change in the direction of the relationship between recent (past week) and past (week before last) injection and naloxone possession or carriage. Rather, the strength of the effect increased with past injection producing even greater odds of both outcomes. While the results of these analyses were significant (*p* < .001), the confidence intervals were very wide suggesting instability in the estimate. We thus elected not to focus on this measure in the interpretation of data. We leave it in the model as a means to control for usual behavior.

## Discussion

This study used a novel EMA approach to measure and explore contextual factors impacting naloxone possession and carriage among PWUO living in NYC. Data collection coincided with the COVID-19 pandemic which allowed us to frame this research with COVID mitigation-related timeframes as a main effect. Our findings paint a relatively positive picture of naloxone possession and carriage over time; with participants consistently reporting both behaviors weekly.

Surprisingly, the odds of consistent possession and carriage were lower before the COVID-related shutdowns compared to during them. This finding is supported by other work by our team that found the proportion of protected opioid use events (those where a witness and naloxone were both present) increased during the first 12-months of COVID-19 in NYC [22]. It could be the case that the implementation of COVID-19 mitigation efforts (e.g., staying at home) and/or warnings about potential disruptions to the drug market influenced PWUO to remain equipped with naloxone during this chaotic time [22, 27, 28].

In this sample, participants who reported a history of overdose had higher odds of consistent naloxone possession. This is an encouraging finding in an era of volatile opioid drug markets, where unintentional overdose continues unabated, and research indicates experiencing a non-fatal overdose is a strong predictor of a subsequent fatal opioid overdose [29]. However, naloxone possession is only relevant when there is another person present to administer it [30]. While participants in our study rarely endorsed burnout or leaving naloxone behind for reasons related to the unwanted burden of responding to an overdose (e.g., feeling responsible for someone else), other studies report it can be emotionally taxing and may lead to cutting social ties [31]. Taken together, this research supports a growing body of literature underscoring the need for social support among people who use opioids because they are likely to be the first to respond during an overdose [32].

In addition, recent injection drug use increased the odds of consistent naloxone carriage. It could be the case that people who inject drugs have greater awareness of their opioid overdose risk, compared to those using other routes of administration, and that they are more likely to receive overdose prevention messaging at SEPs, where naloxone is frequently distributed [33, 34]. Notably, harm reduction service delivery was in flux during COVID, especially during the “shutdowns.” At the same time, drug supply was disrupted, and overdoses were on the rise [35]. It could be the case that PWUO were more likely to hold on to, and carry naloxone, because they recognized the increased likelihood of needing to deploy it [14].

As has been seen in other studies, race, ethnicity, and gender emerged as important determinants of consistent naloxone possession and carriage [36]. Specifically, white and female participants had greater odds of both behaviors. Racially disproportionate access to naloxone may partially explain why overdose-related mortality rates are increasing rapidly among Black and Hispanic/Latinx men [37]. In one small study among patients who recently initiated MOUD, racial/ethnic minority patients had approximately 10 times greater odds of reporting disruptions in naloxone access during the early months of COVID-19 [38]. For NYC in particular, the history of stop-and-frisk practices by police targeting male racial minorities [39] and the criminalization of drug paraphernalia across the US [40] may also contribute to the gender and racial disparities in overdose death. These policies may deter Black and Hispanic/Latinx people from carrying Naloxone and accessing SEPs, thereby restricting community coverage, due to fear of police encounters and arrest [41, 42].

One evidenced-based strategy that relies less on the individual is the strategic placement of public health vending machines that dispense harm reduction supplies (e.g., sterile syringes and condoms) [43]. Pilot research suggests vending machines dispensing naloxone are associated with immediate reductions in opioid-related overdose deaths [44]. Because vending machines have been shown to improve temporal and geographic access to harm reduction supplies and reach individuals who do not access SEP [44], they could be strategically installed to address gender and racial disparities in naloxone access. Other key strategies being tested include naloxone distribution through mail-based and peer delivered distribution [5, 45].

Finally, from our descriptive analysis, we observed the important role of stigma as a barrier to naloxone possession. Not wanting to be perceived as a drug user was reported in nearly one third of EMAs when capturing information on the rationale for inconsistent possession. Stigma can be deadly among PWUO and has been associated with non-fatal overdose and behavior that increases overdose risk [46–48]. This finding highlights the urgent need for interventions to reduce stigma as a barrier to naloxone engagement among PWUO. One previously recommended approach is the incorporation of brief, destigmatizing messages into outreach and overdose education efforts [48]. There are also calls from PWUO and departments of health, among others, for messaging that normalizes naloxone possession while encouraging community-wide empathy for people who use drugs [49]. Supporting lay responders and destigmatizing drug use can help ensure more people who use opioids never use drugs alone and, it will facilitate diffusion of naloxone through social networks more effectively.

This study is not without limitations. First, while we collected a large volume of data over 24-months, this sample of people who use opioids was recruited from a single city with legally sanctioned syringe and naloxone access laws. Moreover, the NYC Department of Health has a robust naloxone distribution program, providing free naloxone at SEPs and hospitals. Second, slightly more than half of participants were unhoused at the time of enrollment which could translate to greater instability and an inability to hold on to naloxone once possessed. However, housing status was not significantly associated with either outcome which suggests this potential selection bias may not be critical for interpreting our findings. We also recognize the sample size of participants is small; however, the unit of analysis in this study–the EMAs themselves–have ample power to detect variations in behavior using mixed effects models that account for the number of data points contributed by each subject. It is standard in EMA research to have fewer participants than actual data point [19]. A notable strength of this paper is the use of EMA, a method that increases ecological validity by using shorter recall periods which improves the accuracy of self-report of both the behavior itself and the situational context associated with its performance (or not).

## Conclusions

Unprecedented opioid overdose mortality in the United States and around the globe signals an urgent need to optimize naloxone access and carriage, even in communities where naloxone is readily available at no cost [9]. Using EMA, an approach that facilitates the collection of event- and person-level behaviors over time, we were able to critically examine naloxone possession and carriage in NYC during the height of the global COVID-19 pandemic. While our findings are encouraging, they also highlight the urgent need for greater community coverage of naloxone in NYC, despite its robust harm reduction infrastructure. Additional low threshold harm reduction and naloxone access points throughout NYC neighborhoods are needed. Moreover, increasing outreach and naloxone distribution to Black and Latinx PWUO is needed to address growing overdose disparities.
